# Barriers and facilitators of integrating physiotherapy into primary health care settings: A systematic scoping review of qualitative research

**DOI:** 10.1016/j.heliyon.2023.e20736

**Published:** 2023-10-06

**Authors:** Shabnam ShahAli, Saeed Shahabi, Manal Etemadi, Maryam Hedayati, Barth Cornelia Anne, Parviz Mojgani, Masoud Behzadifar, Kamran Bagheri Lankarani

**Affiliations:** a Iranian Center of Excellence in Physiotherapy, Rehabilitation Research Center, Department of Physiotherapy, School of Rehabilitation Sciences, Iran University of Medical Sciences, Tehran, Iran; b Health Policy Research Center, Institute of Health, Shiraz University of Medical Sciences, Shiraz, Iran; c The National Institute for Health and Care Research Applied Research Collaboration West (NIHR ARC West), University Hospitals Bristol and Weston NHS Foundation Trust, UK; d Population Health Sciences, Bristol Medical School, University of Bristol, UK; e Department of Health Services Management, School of Health Management and Information Sciences, Iran University of Medical Sciences, Tehran, Iran; f School of Public Health, Physiotherapy and Sports Science, University College Dublin (UCD), Dublin, Ireland; g Centre for Primary Care and Public Health (Unisanté), University of Lausanne, Lausanne, Switzerland; h Iran-Helal Institute of Applied Science and Technology, Tehran, Iran; i Research Center for Emergency and Disaster Resilience, Red Crescent Society of The Islamic Republic of Iran, Tehran, Iran; j Social Determinants of Health Research Center, Lorestan University of Medical Sciences, Khorramabad, Iran

**Keywords:** Physiotherapy, Primary care, Primary health care, Rehabilitation, Qualitative evidence, Scoping review

## Abstract

**Purpose:**

This scoping review investigated the barriers and facilitators to integrating physiotherapy into primary health care (PHC).

**Materials and methods:**

PubMed, Scopus, Web of Science, Embase, ProQuest, and REHABDATA were searched. Two independent reviewers were involved in screening, selecting, and extracting data. Data were synthesized using thematic analysis.

**Results:**

Of the 483 screened documents, 44 qualitative studies, primarily from high-income countries, were included. All of the studies had good methodological quality. Barriers and facilitators of integrating physiotherapy into PHC were extracted within the WHO six building blocks framework. In total, 41 items were identified as barriers to the integration process. The studies included 49 recommendations to facilitate integrating physiotherapy services into PHC.

**Conclusion:**

Integrating physiotherapy services into PHC faces many barriers. The most commonly suggested potential barriers are poor knowledge of physicians about physiotherapy, ineffective teamwork, physiotherapists’ time constraints/workload, a lack of clarity over the role and knowledge of physiotherapists, unawareness of physiotherapy users about these services, and lack of intra- and inter-professional collaborations. The most commonly suggested recommendations to facilitate the integration process include: Clarifying the role of involved professionals, strengthening teamwork, improving intra- and inter-professional collaborations, and providing comprehensive training programs for physiotherapists.

## 1 Introduction

To promote health and well-being for people who experience chronic diseases, the provision of rehabilitation (including physiotherapy) must be considered throughout the health care system [1]. For people with disabilities, rehabilitation services must be explicitly included within essential healthcare services and consideration of the achievement of universal health coverage (UHC) [1,2]. Greater access to rehabilitation is a requirement for achieving SDG 3 on health and will benefit the entire population, not just people with disabilities [3,4].

Primary care is the first point of contact in the health system, where services are mobilized and coordinated to promote health, prevent illness, and manage chronic illness. The future of primary care lies in its ability to meet the needs of people who are chronically ill [5]. Primary care services focus on treating a broad range of health conditions, with an emphasis on health promotion and disease prevention [6]. Primary health care (PHC) requires a multidisciplinary approach, integrating different types of health care professionals, such as physicians, nurses, pharmacists, dieticians, social workers, and rehabilitation professionals [7,8].

Global demographic and health trends will probably increase the need for rehabilitation to be integrated into PHC. Despite the Declaration of Alma-Ata and the existence of successful integration models, in the vast majority of health systems, rehabilitation has not been fully or effectively integrated into PHC [9]. Rehabilitation provided close to people's homes enables them to remain in educational programs and lead productive lives. It minimizes the care and financial support needed, leading to associated benefits for both the individual and society. It can also help to avoid costly hospitalizations and re-admissions [[10], [11], [12]]. The extent to which rehabilitation is timely and delivered along a continuum with effective referral practices, as seen in PHC, is considered an indicator of rehabilitation quality [13].

An important component of rehabilitation is physiotherapy, which should form an integral part of the services offered at the PHC level [14]. Physiotherapists play an important role in reducing musculoskeletal disorders in the community through primary health services [15]. Physiotherapy is recommended to be included in PHC to expand access and provide comprehensive care to the population. The predominance and multi-causality of chronic health conditions and their repercussions on the functional capacity of individuals reinforce the importance of including physiotherapists in PHC policies [16]. The evolving primary care practice in physiotherapy is a direct consequence of the first contact/direct-access privilege in physiotherapy. Because of the quick-access philosophy of PHC delivery, the involved physiotherapists should be able to see the patient on a first-contact basis [17]. Integrating physiotherapists into primary care settings would improve the coordination of care for people with chronic diseases because locating physiotherapists and family physicians in the same working environment enhances interprofessional collaboration through formal and informal communications [18].

Increasing access to chronic disease prevention and management by embedding physiotherapists in PHC settings might be a cost-effective and value-based strategy for providing more tailored, comprehensive, and holistic care [19]. In a PHC team, physiotherapists can assume several roles: they can work with patients with musculoskeletal and neurological conditions, provide fall prevention training, and educate patients and caregivers about preventing and managing chronic diseases [20]. Physicians and nurses valued the integration of physiotherapists into their teams, and physiotherapy was the most requested rehabilitation service [21].

As part of 10.13039/100012453PHC, healthcare education needs to be reoriented towards empowering the client, consumer partnerships and participation, self-care, supporting the person to achieve their health goals, as well as teamwork and community collaboration [22]. Universities should encourage graduates not only to think and practice in their chosen field and discipline but can also to develop beyond those defined abilities. Physiotherapy education must be reimagined to meet workforce demands and address these expectations [17,23]. Knowing the barriers and facilitators for integrating physiotherapy into primary health care settings can facilitate decisions regarding needed changes in the curriculum content and teaching methods.

Until now, several studies have been conducted related to the integration of physiotherapy services into PHC, especially in high-income countries. However, to the best of our knowledge, no study has been conducted to review the findings of these studies. Furthermore, a major proportion of studies have focused only on one aspect of the challenges of integrating physiotherapy services into PHC. In response, the research team has tried to synthesize the findings of the conducted studies using the conceptual framework of the World Health Organization in the field of health policy and, by aggregating and summarizing the barriers and facilitators, provide a suitable platform for the decision makers on the path of integrating physiotherapy services into PHC.

## 2 Methods

This systematic scoping review was developed and written in accordance with the Preferred Reporting Items for Systematic Reviews and Meta-analyses Extension for Scoping Reviews (PRISMA-ScR) checklist [24]. The research team selected scoping review design because of its methodology, which can be especially helpful for bringing together literature in a specific discipline with emerging literature and addressing a wider research question [25,26]. Indeed, scoping reviews are particularly valuable as a kind of evidence synthesis to prepare an opportunity to investigate key themes and concepts. This systematic scoping review was conducted based on Peters et al.'s (2015) guidance [26]. The Institutional Review Board of Shiraz University of Medical Sciences had previously reviewed and approved the study's protocol.

### 2.1 Search strategy

The SPIDER (Sample, Phenomenon of Interest, Design, Evaluation, and Research Type) search framework was applied to develop the search strings [27]. In this regard, two components of this framework, including phenomena of interest (physiotherapy and PHC) and research type (qualitative study), were considered. Based on the evidence, this search framework is more sensitive and reliable than some other frameworks for searching qualitative research [27]. To find related terms, the Medical Subjects Headings (Mesh) thesaurus was scanned. In addition, contacting relevant experts and applying the free-text method were used to cover other potential terms. In total, keywords such as “Primary health care”, “Primary care”, Physiotherapy, “Physical therapy”, Physiotherapist, Physiotherapists, “Physical therapist”, “Physical therapists”, “Qualitative study”, challenge, issue, drawback, and opportunity were used. The initial search strategy was developed for the PubMed database and then modified for other databases (Supplementary Table 1).

From its inception to the end of June 2022 (updated until June 2023), several bibliographic databases were searched structurally, including PubMed, Scopus, Web of Science, Embase, ProQuest, and REHABDATA. Further, Google Scholar, Microsoft Academic, and OpenGrey were searched to curb the potential publication bias. Relevant key journals such as the Journal of Physiotherapy, Physiotherapy Theory and Practice, Disability and Rehabilitation, Physiotherapy Canada, BMC Health Services Research, Scandinavian Journal of Primary Health Care, Australian Journal of Health Care, Journal of Primary Health Care, Qualitative Health Research, Qualitative Research, and others, as well as reference lists and citations from included studies, were manually searched to identify any missed study.

### 2.2 Selection of studies

All the results obtained from the search were entered into the Endnote X8 software (Thomson Reuters, New York, NY), and after removing duplicates, the remaining studies were screened based on the title and abstract. Potentially relevant studies were then reviewed based on the full text, and the final studies were selected for inclusion. These steps were performed by two authors independently, and any disagreement between them was resolved through discussion and the participation of the third author.

### 2.3 Inclusion and exclusion criteria

This review included qualitative studies that investigated the perspectives and experiences of policy-makers, administrators, physiotherapists, and other involved professionals regarding the main barriers and facilitators of integrating physiotherapy services into PHC. The inclusion criteria were: 1) scientific studies with a qualitative design; 2) studies published in a peer-reviewed journal; 3) studies in English; 4) studies exploring the experiences of different stakeholders regarding integrating physiotherapy services into PHC; and 5) availability of the full text of the study. The exclusion criteria were: 1) quantitative studies; 2) non-English language studies; 3) protocol studies, letters to the editor, abstracts, editorials, and comments; 4) review studies; 5) studies without available full text; and 6) qualitative studies that did not focus on the knowledge of experiences related to the integration of physiotherapy services into PHC.

### 2.4 Data extraction

Two authors independently performed the data extraction process. Before starting this process, a data collection form was developed with the participation of all team members, the items of which were: 1) first author; 2) publication year; 3) participants; 4) sampling approach; 5) country of origin; 6) data collection method; 7) interview format; 8) analysis approach; 9) challenges of integration; 10) facilitators of integration; 11) summary of findings; and 12) funding source. Two other authors reviewed this procedure to ensure the accuracy of the extracted data. Any disagreement was resolved at this stage, as it had been in previous stages, through discussion and, in some cases, the participation of the expert author.

### 2.5 Methodological quality evaluation

The Qualitative Checklist of the Critical Appraisal Skills Programme (CASP) tool, which is frequently used to evaluate the methodological quality of qualitative evidence [28], was applied. This tool includes 10 questions in three main sections: (a) Are the results valid? (b) What are the results? and (c) Will the findings be useful locally? Three authors conducted the quality assessment by answering 10 items of the CASP independently. "Yes" (met the item), "no" (did not meet the item), and "unclear/cannot tell" (if the item was not clear) were the possible answers. When there was a disagreement among the involved authors, and they were not sure about the answer provided, meetings were held to discuss and reach an agreement.

### 2.6 Data synthesis and analysis

A thematic analysis approach was utilized to synthesize the collected data from the included qualitative studies [29]. Textual summaries were developed regarding each dimension of the WHO Six Building Blocks Framework [30]. After that, three authors reviewed and evaluated the similarities and differences among these summaries and identified the main themes. The identified themes, including barriers and facilitators, were then grouped and assigned to each dimension of the WHO Six Building Blocks Framework (including stewardship, service delivery, financing, human resources, information systems, and medicine/technologies). This framework was chosen because it has the simplicity and capability to create a common language among researchers and helps to analyze and categorize findings, discuss barriers and facilitators, and inform policy- and decision-makers [30].

## 3 Results

**Fig. 1:**
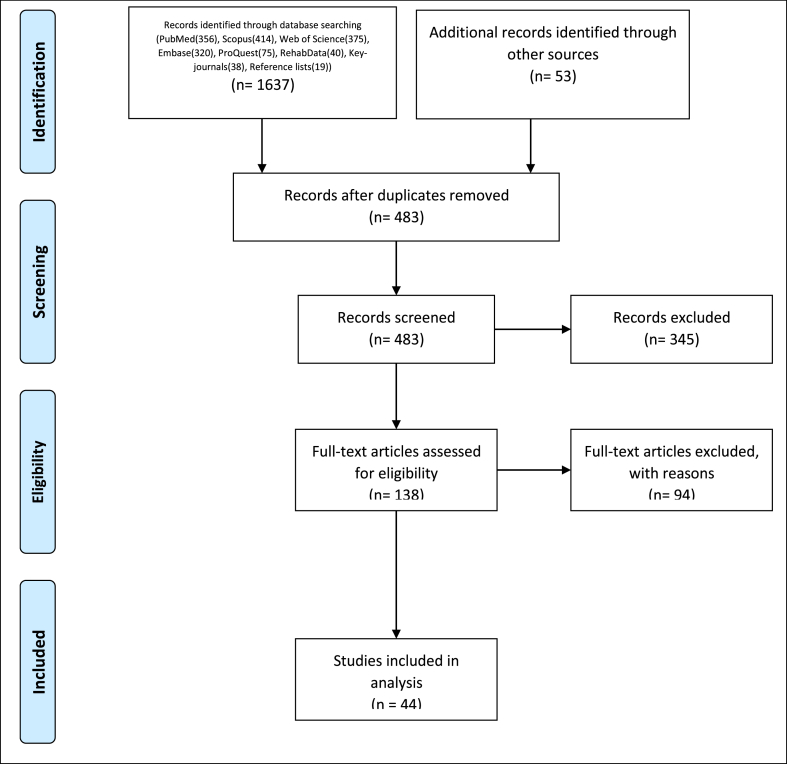
PRISMA flowchart.

The initial search of databases of interest yielded 1690 records. After removing duplicates, 483 studies were screened. Next, 138 studies were evaluated based on their full text, and in the end, 44 qualitative studies [7,16,20,23,[31], [32], [33], [34], [35], [36], [37], [38], [39], [40], [41], [42], [43], [44], [45], [46], [47], [48], [49], [50], [51], [52], [53], [54], [55], [56], [57], [58], [59], [60], [61], [62], [63], [64], [65]] were included in the study. Fig. 1 demonstrates the PRISMA flow chart, indicating the search results and study selection process. A list of 94 excluded studies, along with the reason for exclusion, has been shown in Supplementary Table 2.

### 3.1 Characteristics of included studies

This systematic review included 44 qualitative studies conducted in 14 countries, mostly high-income countries: United Kingdom (27.2 %, n = 12) [34,36,39,47,49,52,58,62,[64], [65], [66], [67]], Sweden (13.6 %, n = 6) [38,43,53,54,59,63], Ireland (11.3 %, n = 5) [32,35,40,44,48], Canada (11.3 %, n = 5) [7,20,23,37,68], the Netherlands (9.1 %, n = 4) [31,42,46,50], Norway (6.8 %, n = 3) [33,41,61], South Africa (4.5 %, n = 2) [60,69], Brazil (2.2 %, n = 1) [16], Chile (2.2 %, n = 1) [45], Germany (2.2 %, n = 1) [56], Iran (2.2 %, n = 1) [51], Spain (2.2 %, n = 1) [55], Australia (2.2 %, n = 1) [70], and multiple-countries (2.2 %, n = 1) [57]. Semi-structured interviews, focus group sessions, or both of them were the common data collection methods, respectively. Regarding the sampling strategy, the purposive approach was the dominant strategy to recruit participants (n = 20). However, six of the included studies [31,33,38,46,48,52] did not mention the sampling method. Thematic and content analysis were the primary analysis approaches among the included studies. Framework analysis, grounded theory, three-stage method, discourse analysis, and the Colaizzi approach were other used methods. Table 1 demonstrates the characteristics of the included studies in detail.

**Table 1:** The characteristics of included studies.

Author (publication year)	Study aim	Participants (N)	Sampling	Country of origin	Data collection method	Interview format	Analysis approach	Summary of findings	Funding source
Åkesson et al. (2021)	Exploring Physiotherapists' Experiences of osteoarthritis guidelines in primary health care	Physiotherapists	Purposive sampling	Sweden	Semi-structured interview	Face-to-face	Content analysis	The physiotherapists were confident in their role as primary assessors for patients with osteoarthritis and the guidelines were aligned with their professional beliefs.	Lund University & Region Skåne
AL Zoubi et al. (2019)	Identify and contrast barriers and facilitators to using the stratified care approaches for NSLBP among physiotherapists and chiropractors	Physiotherapists and chiropractors	Purposive and snowball sampling	Canada	Semi-structured interview	Telephone	Content analysis	Unique key domains were identified among physiotherapists: incompatibility with achieving other objectives, and chiropractors: confidence in using stratified care approaches; intention to use stratified care approaches ; awareness and agreement with stratified care approaches; assessment of readiness for change and planning behavior; and improving the management of nonspecific LBP patients and the uptake of evidence-based practice	Edith Strauss Rehabilitation KT Research Projects
Bassett & Jackson (2020)	Exploring the perceived challenges and learning opportunities of pre-registration physiotherapy placements in musculoskeletal first-contact physiotherapy settings from First-contact physiotherapists' perspectives.	Physiotherapists	Snowball sampling	United Kingdom	Semi-structured interviews	Telephone	Framework analysis	Three core themes emerged: operational challenges, challenges, and learning opportunities for pre-registration physiotherapy students	Health Education England
Bim et al. (2021)	To understand the routine and tools used by physiotherapists in primary health care and analyze the determining factors	Physiotherapists	Convenient sampling	Brazil	Semi-structured interviews	Face-to-face	Discourse analysis	The main tools routinely used in the physiotherapy service were individual appointments, home visits, and group work. Physiotherapy practices were influenced by public health, municipal management and BHU policies, physiotherapy profile in addition to the characteristics of the coverage area and the population being treated	NI
Carlfjord et al. (2018)	Exploring the practitioners' experiences from a structured implementation of an evidence‐based assessment and treatment program for patients with subacromial pain	Physiotherapists	NI	Sweden	Focus group sessions	Face-to-face	Content analysis	The practitioners' experiences from the implementation were mainly positive. A strategy with collaboration between academy and practice, and with education and implementation teams as facilitators, resulted in changes in practice.	NI
Cerderbom et al. (2020)	Exploring physical therapists' view of how they experience and perceive their role in working with fall prevention in a community care setting	Physiotherapists	NI	Norway	Semi-structured interviews	Face-to-face	Thematic analysis	The findings indicate that the physiotherapists' role reflects their abilities to change and improve their professional work by evidence-based knowledge	OsloMet – Oslo Metropolitan University
Cott et al. (2011)	Exploring the potential for different models of incorporating physiotherapy services within the emerging network of family health teams	Individuals who oversaw the overall operations of the family health teams and community-based physiotherapy clinics.	Snowball sampling	Canada	Semi-structured interviews	Face-to-face or by telephone	NI	Most participants agreed that the ideal model involves embedding physiotherapists directly into family health teams; in some situations, however, partnering with existing external physiotherapy, the provider may be more feasible and sustainable	Ontario Ministry of Health and Long-Term Care
Dikkers et al. (2016)	Exploring the barriers and facilitators affecting the implementation of manual therapy in neck pain management in primary care	GPs, physiotherapists, manual therapists, and patients with neck pain	NI	Netherlands	Focus group sessions	Face-to-face	Thematic content approach	Barriers and facilitators were found especially in individual perceptions of manual therapy for neck pain, the interaction between stakeholders and the organizational context	Netherlands Organization for Health Research and Development
Dufour et al. (2014)	To understand physiotherapists' roles and how they are enacted within primary health care teams	Physiotherapists	Purposive and theoretical sampling	Canada	Semi-structured interviews	Face-to-face or by telephone	Grounded theory	physiotherapists carry out multiple roles that are based on a broad holistic perspective of health, within the context of a collaborative inter-professional team and the community, through an evidenced-informed approach to care	NI
French & Galvin (2016)	To explore physiotherapists' experiences of providing musculoskeletal physiotherapy in primary care to gain an insight into their changing roles, challenges in service delivery and continuing professional development	Physiotherapists	NI	Ireland	Focus group sessions	Face-to-face	Thematic analysis	Considerable variation exists in the provision of physiotherapy, and continuing professional development need	Irish Society of Chartered Physiotherapists
French & Galvin (2018)	To explore physiotherapy managers' experiences of managing musculoskeletal physiotherapy services in primary care to gain an insight into the opportunities and challenges in service delivery, changing roles, and ongoing professional development needs of staff	Physiotherapists	Purposive sampling	Ireland	Semi-structured interviews	Face-to-face	Thematic analysis	Several factors that impact on musculoskeletal service delivery in primary care from the perspective of physiotherapy managers were identified	Irish Society of Chartered Physiotherapists
Goodwin et al. (2021)	To report the qualitative findings from the FCP National Evaluation (Phase 3) which evaluated the FCP model against pre-agreed success criteria	GPs, physiotherapists, patients, and administration staff	Purposive sampling	United Kingdom	Semi-structured interviews and focus group sessions	Face-to-face	Thematic analysis	The results demonstrated success in all of the service aims and success criteria. FCP was well received by staff and patients alike	The chartered society of Physiotherapy CSP charitable trust, the Department of Health, and Social care.
Greenhalgh et al. (2020)	To explore the experiences of FCPs to gain insight into the first point of contact service, and their experiences of this developing full-time FCP role	FCPs	Convenient sampling	United Kingdom	Semi-structured interviews	Face-to-face	NI	Most clinicians considered the role an exciting and positive development for the profession, that benefited the patient and the NHS in terms of quality of care and efficiency. However, to realize these benefits, the participants highlighted several issues that require further consideration	The Chartered Society of Physiotherapy
Igwesi-Chidobe et al. (2021)	To understand the experiences of health care professionals and patients on direct access in a region in England with commissioned	GPs, physiotherapists, patients, and clinical commissioners	NI	United Kingdom	Semi-structured interviews	Face-to-face and telephone	Thematic analysis	Direct access to NHS musculoskeletal physiotherapy is acceptable to patients and healthcare professionals	Versus Arthritis
Ingram et al. (2023)	To explore the experiences of uncertainty amongst musculoskeletal FCPs working in primary care	Physiotherapists	Purposive sampling	United Kingdom	Semi‐structured interviews	Zoom video‐ Conferencing platform.	Thematic analysis	Five themes were identified: role clarity within primary care, burden of responsibility, preparedness for the primary care environment, ‘I'm not sure how long I am going to stay in this role, mitigating uncertainty.	No fund
Irgens et al. (2018)	To investigate how physiotherapists experience the way patient information is communicated across health care levels in ABI rehabilitation.	Physiotherapists and patients with ABI	Convenient sampling	Norway	Interview	Face-to-face	Systematic text condensation analysis	The findings indicate the need to improve routines for the communication of information and to clarify issues related to the economy and responsibilities	Norwegian Fund for Post Graduate Training in Physiotherapy; and the Centre for Care Research, North Norway
Karstens et al. (2015)	To explore the views and perceptions of GPs concerning using stratified primary care for LBP	GPs	Convenient sampling	Germany	Focus group sessions	Face-to-face	Content analysis	The attitudes of GPs towards stratified primary care for LBP indicated positive support for pilot testing. However, there were mixed reactions to the ability of physiotherapists manage high-risk patients and handle their complex clinical needs. GPs also mentioned practical difficulties in providing extended advice to low-risk patients	The young scientists program of the German network 'Health Services Research Baden-Württemberg' of the Ministry of Science, Research, and Arts in collaboration with the Ministry of Employment and Social Order, Family, Women and Senior Citizens, Baden-Württemberg
Knoop et al. (2022)	To explore the experiences with stratified exercise therapy from the users of this intervention	Physiotherapists, patients, and dieticians	NI	Netherlands	Semi-structured interviews	Telephone	Thematic analysis	The results revealed several barriers to the effective application of the stratified exercise therapy, especially for the obesity subgroup	The Scientific Board Physical Therapy of the Royal Dutch Society for Physical Therapy
Lewis & Gill (2023)	To explore the experiences of FCPs in primary care in Wales regarding the implementation, interprofessional collaboration, and the facilitators and barriers to providing the service	Physiotherapists	Purposive sampling	United Kingdom	Semi-structured interviews	Face-to-face, virtual	A three-stage method	Findings suggest that FCPs were often acting as the second contact, with long waiting lists, is present in certain models and therefore not saving GP appointment time, as was their intended purpose	No fund
Mackenzie & Clifford (2018)	To explore the perceptions of primary health staff about falls prevention in their practice	GPs, occupational therapists and physiotherapists	Purposive sampling	Ireland	Semi-structured interviews	Face-to-face or by telephone	Thematic analysis	Two key themes emerged from the data: the level of primary care team integration and the nature of community fall prevention, linked by referral mechanisms	No fund
Macpherson et al. (2023)	To explore the current referral practices of recent graduates and experienced physiotherapists who manage people with musculoskeletal conditions and their opinions about a referral to specialist physiotherapists for people at risk of poor outcomes	Physiotherapists	Purposive sampling	Australia	Semi-structured interviews	A videoconferencing platform	Content analysis	Referral practices were influenced by specific diagnoses, the complexity of presentations, confidence, self-awareness, the clinical environment, and system-related factors	One of the authors was supported by a NHMRC Career Development Fellowship
Maharaj et al. (2018)	To gain insight into physiotherapists' perspectives on the perceived barriers and facilitators of integrating physiotherapists into primary health care teams	Physiotherapists	Convenient sampling	Canada	Semi-structured interviews	Face-to-face or by telephone	Thematic analysis	The key findings of influential factors for integration were (1) the diversity and novelty of new physiotherapists The role, (2) team members’ understanding of the physiotherapists' role, and (3) physiotherapists' actions and values regarding primary health care	NI
McCreesh et al. (2016)	To elicit the motivators, barriers, and benefits of participation in a Community of Practice for primary care physiotherapists	Physiotherapists	Convenient sampling	Ireland	Semi-structured interviews	Telephone	Thematic analysis	The majority of participants reported positive clinical practice changes in terms of improved patient education, increased confidence, and availability of new resources.	Health Research Board of Ireland
McDaid et al. (2017)	To explore physiotherapists' views and experiences of physiotherapy service provision for PWNC in primary care	Physiotherapists	Purposive sampling	Ireland	Semi-structured interviews	Face-to-face	Thematic analysis	Physiotherapists identified a mismatch between their ideal service standard and the practice reality of physiotherapy service provision for PWNC	No fund
McMahon et al. (2016)	To explore the perspectives of both national and international physiotherapy educators/practitioners in primary healthcare, on the key elements required in physiotherapy education programs	Physiotherapists	Snowball sampling	Ireland, the United Kingdom, Canada, New Zealand, and Australia	Semi-structured interviews	Telephone	Thematic analysis	The themes identified included; understanding the philosophy of physiotherapy practice, cultural competence, inter-disciplinary team working, and communication skills	NI
Misra et al. (2019)	To explore the experiences of physiotherapy students participating in a CBPHCT platform	Physiotherapy students	Purposive sampling	South Africa	Focus group sessions	Face‐to‐face	Content analysis	Four overarching themes were identified: prerequisite community-based primary healthcare competencies, positive factors associated with CBPHCT, negative factors associated with CBPHCT and recommendations	The College of Health Sciences, Young researcher award at the University of KwaZulu-Natal
Moffatt et al. (2018)	to explore how the professionals and practice staff involved in the delivery of an in-practice physiotherapy self-referral scheme understood the service, with a focus on perceptions of value, barriers, and impact	GPs, physiotherapists, administration/reception staff, one nurse	Purposive sampling	United Kingdom	Individual interviews and focus group sessions	Face‐to‐face	Thematic analysis	Three key themes were highlighted: First, the imperative of effecting a cultural change, Second, the impact of the service on working practice across all Stakeholders, Third, beliefs regarding the nature and benefits of physiotherapeutic musculoskeletal expertise	No fund
Morris et al. (2020)	To explore patient perceived acceptability of the FCP role using realist methods to understand what works for whom, how, why, and in what circumstances	GPs, patients, FCPs, receptionists, and practice managers	Purposive sampling	United Kingdom	Interviews	Telephone and face‐to‐face	Thematic analysis	Patients were predominantly accepting of FCPs, nevertheless, there was a scope to increase acceptability through an implementation strategy that considered the contexts of the individual patient, as well as wider practice contexts	University of the West of England
Narain & Mathye (2023)	To explore strategies to integrate physiotherapy services in primary healthcare settings in South Africa.	Physiotherapists	Purposive sampling	South Africa	Semi-structured interviews	Telephone, Skype	Thematic analysis	Six themes were identified: improve societal knowledge of physiotherapy, ensure policy representation of the profession, transform physiotherapy education, broaden the role of physiotherapy, eradicate professional hierarchy, and increase the physiotherapy workforce.	National research foundation
Neil Langridge (2019)	To understand some of the key skills, knowledge, and attributes used by advanced practice physiotherapists working within a GP setting as a first‐contact practitioner	GPs and physiotherapists	Purposive sampling	United Kingdom	Individual interviews and focus group sessions	Skype	Thematic analysis	The themes identified were: medical assessment and systems knowledge; speed of thought in an uncertain environment; breadth of knowledge; people and communication skills; common sense/simplify; and responsibility and experience	NI
Okwera & May (2018)	To explore the beliefs of GPs on the physiotherapy management of lower limb OA in primary care	GPs	Systematic sampling	United Kingdom	Semi-structured interviews	NI	Content analysis	GPs who were interviewed had a limited understanding of the role of physiotherapists and of clinical guidelines. Inter-professional communication was not as good as it should have been.	No fund
Patel et al. (2014)	To explore healthcare professionals' views on a group-based exercise An intervention designed to facilitate the self-management of OA in the lower limbs and/or lower back	GPs, physiotherapists, and one community-based rheumatologist	Convenient sampling	United Kingdom	Semi-structured interviews	Telephone and face‐to‐face	Thematic analysis	Healthcare professionals saw the intervention as an acceptable and feasible approach to facilitate the self-management of OA	Chartered Society of Physiotherapy Charitable Trust
Paz-Lourido & Kuisma (2013)	To explore the educational factors that underlie the poor collaboration between GPs and physiotherapists in Primary Health Care from the GP's perspective	GPs	Purposive sampling	Spain	In-depth interviews	NI	Discourse analysis	The perceived lack of knowledge about physiotherapy was considered by the interviewees as a major factor in the current poor communication between GPs and physiotherapists Collaboration was considered beneficial for patients but challenging to improve in context due to multiple factors ranging from individual to systemic	University of the Balearic Islands and the Balearic Council for Health Affairs
Pearson et al. (2016)	To explore the acceptability of the PhysioDirect telephone assessment and advice service to patients with musculoskeletal conditions	Physiotherapists	Purposive sampling	United Kingdom	Semi-structured interviews	Face‐to‐face	Thematic analysis	Participants generally viewed both the PhysioDirect service and the physiotherapists providing the service as helpful	Medical Research Council and managed by the National Institute for Health Research
Pellekooren et al. (2022)	To explore the experiences and perceptions of APPs and GPs with respect to implementing the APP	GPs and APPs	Convenient sampling	Netherlands	Semi-structured interviews	Online video call	Thematic analysis	Implementing an APP model of care was challenging, in part, because the deployment of APP did not sufficiently align with the core values of GPs, while GPs appear reluctant to hand	Dutch Association for Manual Therapy
Rasmussen-Barr et al. (2018)	To explore how leadership manifests in the patient-therapist interaction among physiotherapists in primary health care and how the physiotherapists themselves relate their perception of leadership in their clinical practice	Physiotherapists	Purposive sampling	Sweden	Semi-structured interviews	Face-to-face or telephone	Content analysis	Five themes were identified related to how leadership manifests in the patient-therapist interaction: (1) establishing resonant relationships; (2) engaging patients to build ownership; (3) drawing on authority; (4) building on professionalism; and (5) relating physiotherapists' clinical practice to leadership	NI
Reyes et al. (2020)	To describe the perceived quality of physiotherapy care from primary care outpatients	Patients	Purposeful non-probabilistic sampling	Chile	Semi-structured interviews	NI	Colaizzi approach	The quality of physiotherapy care was related to subjective (relational) and objective (Structural) perceptions	No fund.
Shahabi et al. (2022)	Integrating rehabilitation services in primary health care: Policy Options for Iran	Health policymakers, rehabilitation managers, faculty members, and practitioners	Purposive and snowball sampling	Iran	Semi-structured interviews	Face-to-face and telephone	Framework analysis	This study identified some policy options, such as increasing political support; promoting inter-sectoral collaborations; increasing the skills and knowledge of health care workers; effective referral pathways; teamwork; and increasing government funding, for integrating rehabilitation services into the Iranian PHC Network based on the WHO six building blocks framework.	No fund.
Stigmar et al. (2014)	To analyze how a group of experienced and specially trained physiotherapists in primary health care perceived their professional role in work ability assessments	Physiotherapists	Strategic sampling	Sweden	Focus group interviews	Face-to-face	Content analysis	Four categories were agreed upon: the need to emphasize the physiotherapists' role in the organization, the benefits of continuity, to contribute to more structured assessments, and to take more initiative	NI
Tran et al. (2018)	To describe how healthcare students perceived conditions for interprofessional education in primary healthcare	Nursing, physiotherapy, occupational therapy and medicine students	Convenient sampling	Sweden	Focus group interviews	Face-to-face	Content analysis	Findings indicated one theme: Students perceived a need for support and awareness of interprofessional education from both study programs and clinical placements	Karolinska Institute and Stockholm County Council
Vader et al. (2022)	To understand the perspectives of patients and primary care team members related to their experiences with a new physiotherapist-led primary care model for LBP	LBP patients and Primary care team members	Purposive sampling	Canada	Semi-structured interviews and focus group discussions	Telephone	Thematic analysis	Four themes were identified: enhanced primary care delivery for LBP, positive patient experiences and perceived outcomes with the new model of care, positive primary care team experiences with the new model of care, and challenges in implementing the new model of care	Musculoskeletal Health and Arthritis from the Canadian Institutes of Health Research. One of the authors was supported by Frederick Banting and Charles Best Canada Graduate Scholarship from the Canadian Institutes of Health Research, PhD Salary Award from the Arthritis Society Trainee Research Award, and Queen Elizabeth II Graduate Scholarship in Science & Technology
Verwoerd et al. (2022)	To explore physiotherapists' knowledge, attitude, and practice behavior in assessing and managing patients with non-specific, non-traumatic, acute- and subacute neck pain, with a focus on prognostic factors for chronification	Physiotherapists	Purposive sampling	Netherlands	Semi-structured interviews	NI	Content analysis	Seven themes were identified: physiotherapists' self-estimated knowledge and attitude, role clarity, therapeutic relationship, internal and external barriers to practice behavior, physiotherapists' practice behaviors, and self-reflection	Institute of Movement studies and partly by a research voucher from Utrecht University of Applied Sciences
Widerström et al. (2019)	To explore and describe aspects influencing physiotherapists' clinical reasoning in the decision-making on individualized treatment of LBP in primary healthcare	Physiotherapists	Convenient sampling	Sweden	Semi-structured interviews	Face-to-face	Content analysis	Two themes were identified: matching requires differentiation and adaptation, with categories describing specific patient characteristics, assessment findings, and treatment adaptations, the tension between trust and barriers; with categories describing aspects of physiotherapists' convictions, constraints, and working environment	No fund.
Worum et al. (2020)	To explore physiotherapists' perceptions of external factors regarding the relation between knowledge translation and the three elements of evidence-based practice to effectively address barriers and facilitate the uptake of EBP in fall prevention	Physiotherapists	Purposive sampling	Norway	Semi-structured interviews	Face-to-face	Thematic analysis	The findings revealed tension between policy, leadership, organizational facilitators and evidence-based practice. Leadership is influenced by policy with ripple effects for the organization and clinicians.	No fund

ABI = acquired brain injury, APP = Advanced Practitioner Physiotherapy, BHU = basic health unit, CBPHCT = community-based primary healthcare clinical training, NI=No information, FCP= First Contact Physiotherapy, GP= General practitioner, NHS= National Health Service, NSLBP = non-specific low back pain, LBP = low back pain, OA= Osteoarthritis, PWNC = people with neurological conditions.

### 3.2 Methodological quality evaluation

**Fig. 2:**
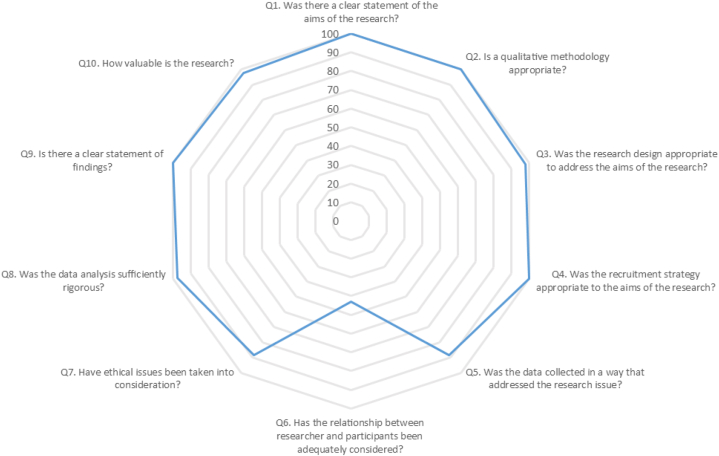
Radar chart of the adherence of included studies to CASP items.

The results of the methodological evaluation of the included studies have been demonstrated in Table 2. All of the studies fulfilled more than 80 % of the CASP questions and had good quality status. However, as shown in Fig. 2, the adherence of included studies to Question 6 (43.2 %), Question 5 (88.6 %), and Question 7 (88.6 %) were the lowest, respectively.

**Table 2:** Summary of the CASP critical appraisal criteria and results.

CASP critical appraisal criteria											
**Q1. Was there a clear statement of the aims of the research?**											
**Q2. Is a qualitative methodology appropriate?**											
**Q3. Was the research design appropriate to address the aims of the research?**											
**Q4. Was the recruitment strategy appropriate to the aims of the research?**											
**Q5. Was the data collected in a way that addressed the research issue?**											
**Q6. Has the relationship between the researcher and participants been adequately considered?**											
**Q7. Have ethical issues been taken into consideration?**											
**Q8. Was the data analysis sufficiently rigorous?**											
**Q9. Is there a clear statement of findings?**											
**Q10. How valuable is the research?**											
**CASP critical appraisal results**											
**References**	**Critical Appraisal Skills Programme (CASP) Qualitative Checklist**										**Quality score**
	**Section A: Are the results valid?**						**Section B: What are the results?**			**Section C: Will the results help locally?**	
	**Q1**	**Q2**	**Q3**	**Q4**	**Q5**	**Q6**	**Q7**	**Q8**	**Q9**	**S10**	
Åkesson et al. (2021)	Y	Y	Y	Y	Y	Y	C/T	Y	Y	Y	9.5/10
AL Zoubi et al. (2019)	Y	Y	Y	Y	Y	Y	Y	Y	Y	Y	10/10
Bassett & Jackson (2020)	Y	Y	Y	Y	Y	Y	Y	Y	Y	Y	10/10
Bim et al. (2021)	Y	Y	Y	Y	Y	Y	Y	Y	Y	Y	10/10
Carlfjord et al. (2018)	Y	Y	Y	Y	Y	C/T	Y	Y	Y	Y	9.5/10
Cerderbom et al. (2020)	Y	Y	Y	Y	Y	Y	Y	Y	Y	Y	10/10
Cott et al. (2011)	Y	Y	Y	Y	C/T	C/T	Y	C/T	Y	Y	8.5/10
Dikkers et al. (2016)	Y	Y	Y	Y	C/T	Y	Y	Y	Y	Y	9.5/10
Dufour et al. (2014)	Y	Y	Y	Y	Y	C/T	Y	Y	Y	Y	9.5/10
French & Galvin (2016)	Y	Y	Y	Y	Y	C/T	Y	Y	Y	Y	9.5/10
French & Galvin (2018)	Y	Y	Y	Y	Y	C/T	Y	Y	Y	Y	9.5/10
Goodwin et al. (2021)	Y	Y	Y	Y	Y	Y	Y	Y	Y	Y	10/10
Greenhalgh et al. (2020)	Y	Y	Y	Y	Y	Y	Y	Y	Y	Y	10/10
Igwesi-Chidobe et al. (2021)	Y	Y	Y	Y	Y	Y	Y	Y	Y	Y	10/10
Ingram et al. (2023)	Y	Y	Y	Y	Y	C/T	Y	Y	Y	Y	9.5/10
Irgens et al. (2018)	Y	Y	Y	Y	Y	Y	N	Y	Y	Y	10/10
Karstens et al. (2015)	Y	Y	Y	Y	Y	C/T	Y	Y	Y	Y	9.5/10
Knoop et al. (2022)	Y	Y	Y	Y	Y	Y	Y	Y	Y	Y	10/10
Lewis & Gill (2023)	Y	Y	Y	Y	Y	C/T	Y	Y	Y	Y	9.5/10
Mackenzie & Clifford (2018)	Y	Y	Y	Y	Y	C/T	Y	Y	Y	Y	9.5/10
Macpherson et al. (2023)	Y	Y	C/T	Y	Y	Y	Y	Y	Y	Y	9.5/10
Maharaj et al. (2018)	Y	Y	Y	Y	Y	Y	Y	Y	Y	Y	10/10
McCreesh et al. (2016)	Y	Y	Y	Y	Y	C/T	Y	Y	Y	Y	9.5/10
McDaid et al. (2017)	Y	Y	Y	Y	Y	Y	C/T	Y	Y	Y	9.5/10
McMahon et al. (2016)	Y	Y	Y	Y	Y	C/T	Y	Y	Y	Y	9.5/10
Misra et al. (2019)	Y	Y	Y	Y	Y	C/T	C/T	Y	Y	Y	9/10
Moffatt et al. (2018)	Y	Y	Y	Y	Y	C/T	Y	Y	Y	Y	9.5/10
Morris et al. (2020)	Y	Y	Y	Y	Y	Y	Y	Y	Y	Y	10/10
Narain & Mathye (2023)	Y	Y	Y	Y	C/T	C/T	Y	Y	Y	Y	9/10
Neil Langridge (2019)	Y	Y	Y	Y	Y	Y	Y	Y	Y	Y	10/10
Okwera & May (2018)	Y	Y	Y	Y	C/T	C/T	C/T	Y	Y	Y	8.5/10
Patel et al. (2014)	Y	Y	Y	Y	Y	C/T	Y	Y	Y	Y	9.5/10
Paz-Lourido & Kuisma (2013)	Y	Y	Y	Y	Y	C/T	Y	Y	Y	Y	9.5/10
Pearson et al. (2016)	Y	Y	Y	Y	Y	C/T	Y	Y	Y	Y	9.5/10
Pellekooren et al. (2022)	Y	Y	Y	Y	Y	C/T	Y	Y	Y	Y	9.5/10
Rasmussen-Barr et al. (2018)	Y	Y	Y	Y	Y	C/T	Y	Y	Y	Y	9.5/10
Reyes et al. (2020)	Y	Y	Y	Y	Y	C/T	Y	Y	Y	Y	9.5/10
Shahabi et al. (2022)	Y	Y	Y	Y	Y	C/T	Y	Y	Y	Y	9.5/10
Stigmar et al. (2014)	Y	Y	Y	Y	C/T	C/T	Y	Y	Y	Y	9/10
Tran et al. (2018)	Y	Y	Y	Y	Y	C/T	Y	Y	Y	Y	9.5/10
Vader et al. (2022)	Y	Y	Y	Y	Y	Y	Y	Y	Y	Y	10/10
Verwoerd et al. (2022)	Y	Y	Y	Y	Y	Y	Y	Y	Y	Y	10/10
Widerström et al. (2019)	Y	Y	Y	Y	Y	C/T	Y	Y	Y	C/T	9/10
Worum et al. (2020	Y	Y	Y	Y	Y	Y	Y	Y	Y	Y	10/10
**TOTAL adherence**	**100 %**	**100 %**	**97.7 %**	**100 %**	**88.6 %**	**43.2 %**	**88.6 %**	**97.7 %**	**100 %**	**97.7 %**	

**Abbreviations:** Y, yes; C/T, Can't Tell; N, no.

### 3.3 Barriers to integrating physiotherapy into PHC

Table 3 outlines the identified barriers to integrating physiotherapy services into PHC settings under the WHO six building blocks framework. Regarding stewardship (leadership/governance), the common barriers were the following: lack of awareness among politicians [41,69]; lack of a national strategy [40,54]; lack of organizational support [16,32,43,48]; lack of intra- and inter-professional collaboration [7,20,38,39,46,59]; ineffective teamwork [32,33,[42], [43], [44],^48^,^52^,^67^]; unclear borders among different professionals [31,48,66]; a lack of comprehensive supervision [46]; inconclusive scientific evidence [31]; and poor standardization of services [40]. Participants in included studies also presented several challenges related to the service delivery process, including 1) lack of clarity over the role and knowledge of physiotherapists [16,20,33,36,39,40,50,55,66,67], 2) poor knowledge of GPs [31,33,39,44,50,55,56,[65], [66], [67]], and 3) unawareness of physiotherapy service users [16,31,37,43,58,59,61,68,69]. In addition, time constraints and workload [31,32,35,37,38,41,50,62,67,68], lack of cohesion and continuity [40,61,62,65], isolation of physiotherapists [7,36,40,66], lack of adherence to clinical guidelines [42,43,52], undesirable referral mechanisms [20,31,40,44,[65], [66], [67]], language barriers [36,37,43], lack of attention to the quality of services [16,32], insufficient follow-up [16,63], complex care needs [32,46,[66], [67], [68]], and long waiting lists [52,67] emerged as the other main challenges of service delivery.

**Table 3:** The barriers to integrating physiotherapy services into primary health care.

WHO six building blocks framework	Categories	Studies
Stewardship (governance/leadership)	Lack of awareness among politicians	(Worum-2020), (Narain & Mathye-2023)
	Lack of a national strategy	(French-2018), (Stigmar-2014)
	Lack of organizational support	(Åkesson-2021), (French-2016), (McDaid-2017), (Bim-2021)
	Lack of intra- and inter-professional collaboration	(Carlfjord-2018), (Cott-2011), (Knoop-2022), (Maharaj-2018), (Tran-2018), (Moffatt-2018)
	Ineffective teamwork	(French-2016), (Åkesson-2021), (Cerderbom-2020), (McDaid-2017), (Igwesi-Chidobe-2021), (McDaid-2017), (Mackenzie-2018), (Verwoerd-2022), (Lewis & Gill-2023)
	Unclear border among different professionals	(Dikkers-2016), (French-2016), (Ingram-2023)
	Lack of comprehensive supervision	(Knoop-2022)
	Inconclusive scientific evidence	(Dikkers-2016)
	Poor standardization of services	(French-2018)
Service delivery	Time constraints and workload	(Carlfjord-2018), (McCreesh-2016), (McDaid-2017), (AL Zoubi-2019), (Bassett-2020), (Dikkers-2016), (Worum-2020), (Pellekooren-2022), (Lewis & Gill-2023), (Vader-2022)
	Lack of cohesion and continuity	(French-2018), (Irgens-2018), (Bassett-2020), (Okwera-2018)
	Lack of clarity over the role and knowledge of Physiotherapists	(French-2018), (Greenhalgh-2020), (Cerderbom-2020), (Maharaj-2018), (Bim-2021), (Pellekooren-2022), (Paz-Lourido-2013), (Moffatt-2018), (Ingram-2023), (Lewis & Gill-2023)
	Isolation of Physiotherapists	(French-2018), (Cott-2011), (Greenhalgh-2020), (Ingram-2023)
	Poor knowledge of GPs	(Cerderbom-2020), (Moffatt-2018), (Pellekooren-2022), (Dikkers-2016), (Karstens-2015), (Okwera-2018), (Paz-Lourido-2013), (Pellekooren-2022), (Mackenzie-2018), (Ingram-2023), (Lewis & Gill-2023),
	Lack of adherence to clinical guidelines	(Åkesson-2021), (Igwesi-Chidobe-2021), (Verwoerd-2022)
	Undesirable referral mechanisms	(Dikkers-2016), (French-2018), (Mackenzie-2018), (Maharaj-2018), (Okwera-2018), (Ingram-2023), (Lewis & Gill-2023)
	Language barriers	(AL Zoubi-2019), (Åkesson-2021), (Greenhalgh-2020)
	Lack of attention to the quality of services	(McDaid-2017), (Bim-2021)
	Insufficient follow-up	(Bim-2021), (Widerström-2019)
	Unawareness of users	(Åkesson-2021), (AL Zoubi-2019), (Goodwin-2021), (Irgens-2018), (Tran-2018), (Dikkers-2016), (Bim-2021), (Narain & Mathye-2023), (Vader-2022)
	Complex care needs	(Knoop-2022), (McDaid-2017), (Ingram-2023), (Lewis & Gill-2023), (Vader-2022)
	Long waiting lists	(Igwesi-Chidobe-2021), (Lewis & Gill-2023)
Financing	Financial hardships	(Bassett-2020), (AL Zoubi-2019), (Dikkers-2016), (Vader-2022)
	Insufficient funding system	(Bassett-2020), (Cott-2011), (French-2016), (French-2018), (Mackenzie-2018), (Macpherson-2023), (Vader-2022)
	Inappropriate payment mechanisms	(Cott-2011)
	Undesirable financing structure	(Knoop-2022), (Pellekooren-2022)
Human resources	Learning challenges	(Misra-2019), (Paz-Lourido-2013), (Paz-Lourido-2013), (Lewis & Gill-2023)
	Educational challenges	(Widerström-2019), (Pellekooren-2022), (Worum-2020), (Misra-2019), (Verwoerd-2022), (Bassett-2020), (Ingram-2023)
	Lack of expertise	(AL Zoubi-2019)
	Poor research skills	(McCreesh-2016), (Worum-2020)
	Having different roles	(French-2016), (French-2018), (Dufour-2014), (Maharaj-2018)
	High workload	(Widerström-2019), (Greenhalgh-2020), (Mackenzie-2018), (Ingram-2023)
	Limited career progression	(French-2018), (French-2016), (Ingram-2023)
	Lack of motivation	(Carlfjord-2018), (Knoop-2022), (Greenhalgh-2020), (Lewis & Gill-2023)
	Lack of confidence	(AL Zoubi-2019), (Okwera-2018), (Pellekooren-2022), (Lewis & Gill-2023), (Macpherson-2023)
	Conflict of interests	(Dikkers-2016)
	Poor staffing	(French-2018), (McDaid-2017), (Mackenzie-2018)
Information systems	Lack of axillary services	(French-2018), (Greenhalgh-2020)
	Lack of consistency among transferred information	(Irgens-2018)
Medicines and technologies	Lack of sufficient equipment	(AL Zoubi-2019), (French-2016), (French-2018), (Maharaj-2018), (McDaid-2017)
	Poor infrastructure	(Bim-2021), (French-2016), (Misra-2019)

In terms of financing, seven studies [7,40,44,48,62,68,70] found inadequate funding systems one of the most critical barriers to integrating physiotherapy in primary care settings. Furthermore, participants in some of the included studies mentioned financial hardships [31,37,62], inappropriate payment mechanisms [7], and undesirable financing structures [46,50] as other financial challenges. Included qualitative studies found several barriers related to human resources, among which the most common ones were: educational challenges [41,42,50,60,62,63,66]; having different roles [20,23,40,48]; and high workloads [36,44,63,66]. Interestingly, lack of motivation [36,38,46,67] and limited career progression [40,48,66] were other issues found related to human resources that could prevent the effective integration of physiotherapy services into primary care. Some challenges have also been raised concerning the fifth dimension of the adapted framework, called information systems. Three studies [36,40,61] presented a lack of axillary services and a lack of consistency among transferred information as the common barriers in this regard. Finally, two challenges were identified as common technology barriers: a lack of sufficient equipment [20,32,37,40,48] and poor infrastructure [16,48,60].

### 3.4 Facilitators of integrating physiotherapy into PHC

Table 4 demonstrates the suggested facilitators for integrating physiotherapy into PHC. Regarding the stewardship dimension, 1) improving intra- and inter-professional collaborations [20,31,32,40,41,43,51,61,65,66]; 2) strengthening teamwork [32,33,35,37,39,48,51,[57], [58], [59],[67], [68], [69], [70]]; 3) effective communication among PHC team members [20,33,34,45,52,57,68,70]; and 4) enhancing patients’ awareness [35,47,49,61,69] were among the most common facilitators provided by the participants in the included studies. 10.13039/100014337Furthermore, some studies stated that employing effective advocacy strategies [20,23,40,45,57,60], gaining political support [51], and increasing the awareness of policymakers [51,69] were necessary prerequisites for the effective integration of physiotherapy services into 10.13039/100012453PHC. Participants in one study [51] believed that the participation of physiotherapists in relevant policymaking processes should be greater. Identifying and involving community engagement [40,51,60], effective communication [20,33,34,45,52,57,68,70], patient-centered planning [45,53,66,68], strengthening scientific evidence [41,50,51,69], and involving the private sector [65,69] were other identified solutions regarding the stewardship component.

**Table 4:** Suggested recommendations to facilitate integrating physiotherapy services into PHC.

WHO six building blocks framework	Recommendations	Studies
**Stewardship**	Employing effective advocacy strategies	(Dufour-2014), (Reyes-2020), (French-2018), (Maharaj-2018), (McMahon-2016), (Misra-2019)
	Increasing political support	(Shahabi-2022)
	Increasing the awareness of policymakers	(Shahabi-2022), (Narain & Mathye-2023)
	Empowering the leadership	(Cerderbom-2020), (Worum-2020), (Shahabi-2022)
	Participation of physiotherapists in Policymaking	(Shahabi-2022)
	Improving intra- and inter-professional collaborations	(Åkesson-2021), (Dikkers-2016), (French-2018), (Maharaj-2018), (Okwera-2018), (Irgens-2018), (Worum-2020), (McDaid-2017), (Shahabi-2022), (Ingram-2023)
	Facilitating the community engagement	(French-2018), (Misra-2019), (Shahabi-2022)
	Strengthening teamwork	(AL Zoubi-2019), (Cerderbom-2020), (French-2016), (Goodwin-2021), (McCreesh-2016), (McDaid-2017), (McMahon-2016), (Moffatt-2018), (Tran-2018), (Shahabi-2022), (Lewis & Gill-2023), (Macpherson-2023), (Narain & Mathye-2023), (Vader-2022)
	Effective communication	(Cerderbom-2020), (Neil Langridge-2019), (Maharaj-2018), (Reyes-2020), (Igwesi-Chidobe-2021), (McMahon-2016), (Macpherson-2023), (Vader-2022)
	Enhancing patients' awareness	(McCreesh-2016), (Morris-2020), (Irgens-2018), (Patel-2014), (Narain & Mathye-2023)
	Patient-centered planning	(Rasmussen-Barr-2018), (Reyes-2020), (Ingram-2023), (Vader-2022)
	Strengthening scientific evidence	(Worum-2020), (Pellekooren-2022), (Shahabi-2022), (Narain & Mathye-2023)
	Involving private sector	(Okwera-2018), (Narain & Mathye-2023)
**Services delivery**	Preparing an appropriate space	(AL Zoubi-2019), (Okwera-2018)
	Direct access to physiotherapy	(Igwesi-Chidobe-2021)
	Regular monitoring	(AL Zoubi-2019), (Stigmar-2014)
	Using web-based services	(Okwera-2018)
	Improving the working culture	(Cerderbom-2020), (McCreesh-2016), (Åkesson-2021), (Rasmussen-Barr-2018), (Worum-2020), (Narain & Mathye-2023)
	Enhancing GPs' awareness	(Moffatt-2018), (Pellekooren-2022), (Goodwin-2021), (Lewis & Gill-2023), (Narain & Mathye-2023)
	Creating a desirable referral system	(French-2018), (Okwera-2018), (Shahabi-2022)
	Adopting bio-psychosocial approaches	(Verwoerd-2022), (Karstens-2015)
	Elevating the pharmacological knowledge of physiotherapists	(McMahon-2016), (Neil Langridge-2019)
	Moving towards professionalism	(Worum-2020), (Rasmussen-Barr-2018)
	Effective communication between patients and physiotherapists	(McDaid-2017), (Rasmussen-Barr-2018), (Igwesi-Chidobe-2021), (Neil Langridge-2019), (Verwoerd-2022)
	Enhancing physiotherapists' capabilities to use EBP	(Worum-2020)
	Structuring the curriculum	(McMahon-2016)
	Clarifying the role of involved professionals	(Maharaj-2018), (French-2016), (Moffatt-2018), (Morris-2020), (Stigmar-2014), (Igwesi-Chidobe-2021), (Mackenzie-2018), (Verwoerd-2022), (Rasmussen-Barr-2018), (Pellekooren-2022), (Shahabi-2022), (Narain & Mathye-2023),
	Make quick decisions	(Neil Langridge-2019)
	Using mobile rehabilitation teams	(Shahabi-2022)
	Using local voluntaries	(Shahabi-2022)
**Financing**	Increasing funding resources	(Goodwin-2021), (Shahabi-2022), (Maharaj-2018)
	Preparing a package of rehabilitation services	(Shahabi-2022)
	Considering competitive payment mechanisms	(Shahabi-2022)
	Reducing out-of-pocket	(Vader-2022)
Human resources	Providing a comprehensive training	(Greenhalgh-2020), (French-2018), (McMahon-2016), (Misra-2019), (Karstens-2015), (McDaid-2017), (Stigmar-2014), (Okwera-2018), (Shahabi-2022), (Ingram-2023), (Lewis & Gill-2023), (Narain & Mathye-2023)
	Facilitating personal and professional development	(French-2016), (McCreesh-2016), (Misra-2019), (Goodwin-2021), (Shahabi-2022), (Lewis & Gill-2023)
	Management and mentorship support	(AL Zoubi-2019), (Greenhalgh-2020), (McDaid-2017), (McCreesh-2016), (Tran-2018), (McMahon-2016), (Macpherson-2023)
	Staff availability	(Dikkers-2016), (French-2018), (Worum-2020), (Shahabi-2022), (Narain & Mathye-2023)
	Increasing awareness of physiotherapists regarding PHC	(Maharaj-2018), (McMahon-2016), (Narain & Mathye-2023)
	Applying staff rotations	(French-2018)
	Being able to work in a flexible manner	(McMahon-2016), (McDaid-2017), (Maharaj-2018)
	Strengthening management skills	(McDaid-2017), (Maharaj-2018)
	Improving clinical and professional competences	(Cerderbom-2020), (Greenhalgh-2020), (French-2018), (Macpherson-2023)
	Teaching the use of information technology to physiotherapists	(McMahon-2016)
	Enhancing Confidence of Physiotherapists	(AL Zoubi-2019), (McCreesh-2016), (Reyes-2020), (Neil Langridge-2019), (Goodwin-2021), (Macpherson-2023)
Health information	Establishing an effective surveillance system	(Shahabi-2022)
	Creating a national quality register	(Åkesson-2021)
	Organizing an electronic information system	(Åkesson-2021), (Karstens-2015)
Medicines and technology	Supplying necessary equipment	(Igwesi-Chidobe-2021), (Shahabi-2022)
	Applying new technological innovations	(French-2018), (Pearson-2016), (Shahabi-2022)

Regarding the service delivery process, the participants of qualitative studies expressed various solutions, among which we can mention improving the working culture [33,35,41,43,53,69], enhancing GPs’ awareness [39,50,58,67,69], creating a desirable referral system [40,51,65], ensuring effective communication between patients and physiotherapist [32,34,42,52,53], and clarifying the roles of involved professionals [20,39,42,44,47,48,[50], [51], [52], [53], [54],^69^]. Besides these, studies stated that a suitable space for physiotherapy practices [37,65] and the possibility of direct patient access to the physiotherapist [52] could be other facilitators. Considering the nature of PHC, the participants emphasized the importance of adopting bio-psychological approaches by physiotherapists [42,56] and having pharmaceutical knowledge [34,57] in the process of service provision. Notably, there were also some solutions to facilitate the provision of physiotherapy services in primary care, especially in remote areas and areas that do not have enough specialist staff, among which the following can be mentioned: using web-based services [65], using mobile rehabilitation teams [51], and using local volunteers [51].

Four qualitative studies [20,51,58,68] also stated solutions related to financing to smooth the integration of physiotherapy services, which included increasing financial resources, developing a package of rehabilitation services including physiotherapy in PHC, considering competitive payment mechanisms to create incentives, and reducing out-of-pocket. Regarding human resources, participants stated that considering a comprehensive training program for physiotherapists [32,36,40,54,56,57,60,[65], [66], [67],^69^], as well as facilitating their personal and professional development [35,48,51,58,60,67], and providing management and mentorship support [32,[35], [36], [37],^57^,^59^,^70^], can significantly improve their participation and performance in 10.13039/100012453PHC. In addition to increasing the awareness of physiotherapists regarding PHC [20,57,69], they need to learn the necessary administrative skills [20,32] and have the ability to work in a flexible manner [20,32,57]. Specifically, in one of the studies, participants stated that staff rotation can be used to familiarize physiotherapists with different clinical environments [40]. Some studies also emphasized the importance of enhancing the confidence of physiotherapists to perform effectively in PHC [34,35,37,45,58,70].

Several solutions related to the fifth dimension of the framework, which is the health information system, were also presented, including establishing an effective surveillance system [51], creating a national quality register [43], and organizing an electronic information system [43,56]. Indeed, participants believed that such information systems could considerably facilitate integrating physiotherapy services into PHC. Finally, participants of the included studies proposed two recommendations regarding the technology dimension, including supplying necessary equipment [51,52] and applying new technological innovations [40,51,64].

## 4 Discussion

According to this scoping review, there are several barriers regarding different dimensions of the WHO six building blocks framework for integrating physiotherapy services into PHC in both well-resourced and less well-resourced countries. On the other hand, a wide range of solutions can significantly facilitate the integration of physiotherapy services into primary care. In the following, we discuss the most important findings of this study.

Physiotherapy services are rarely considered in policy-making processes. This is due to a lack of awareness among politicians and policy-makers [71,72]. Also, physicians tend to focus on medical interventions and neglect other services such as physiotherapy. As a result, many countries have no clear national strategy in relation to the provision of physiotherapy services in PHC [73,74]. Therefore, it is necessary to consider a range of policies, such as greater participation of physiotherapy specialists in policy-making processes as well as holding joint meetings, to increase the awareness of policy- and decision-makers regarding the favorable effects of timely provision of physiotherapy services, especially preventive effects [22,72]. Some studies pointed out the necessity of using advocacy strategies and empowering the leadership of physiotherapy in different countries to facilitate integrating physiotherapy services into primary care. This finding has been emphasized in the study of McColl et al. (2009), which reviewed the available literature regarding integrating rehabilitation services into PHC [75]. To meet this goal, stakeholders such as scientific associations of physiotherapy and service target groups should play a stronger role to gain the support of powerful institutions such as governments, parliaments, and other influential institutions [74,76].

Among others, lack of intra- and inter-professional collaboration and ineffective teamwork were common stewardship-related challenges. Interdisciplinary collaboration and teamwork are crucial, considering the nature of PHC engaging different professions in the service delivery process [77,78]. Lack of interdisciplinary collaboration and ineffective teamwork challenge the provision of these [79]. Such a situation can be seen more acutely among professions that have recently been added to PHC teams, such as physiotherapy [43,48,52]. Factors such as lack of clarity over the role and knowledge of physiotherapists and poor knowledge of other involved professionals, like GPs, contribute to ineffective teamwork [32]. Notably, insufficient cooperation among physiotherapists seen in PHC has challenged their performance as primary therapists [20]. An additional obstacle is the inadequate awareness of service recipients about physiotherapy services, further hampering the provision of these services as primary care. Many patients favor an evaluation and treatment received by a doctor rather than a physical therapist [63,80]. Therefore, it is necessary to use various strategies, such as briefing sessions and awareness campaigns, to increase the awareness of PHC team members and recipients of these services [81].

Another identified challenge was the lack of clear boundaries among professions involved in providing primary care. Previous studies conducted in connection with PHC have also pointed to such an issue, which can cause tension and disagreement among the members of the PHC team [80,82]. In response to such conditions, it is necessary to clarify the role, duties, and abilities of each member of the PHC team by providing the necessary training for the involved professions [51]. By adopting such a strategy, in addition to reducing interprofessional conflicts, necessary action is taken to increase convergence and synergy among participating professions [83]. Furthermore, one of the included qualitative studies cited inconclusive scientific evidence as one of the barriers related to the stewardship dimension that could hamper the process of integrating physiotherapy services [31]. It has been mentioned in previous studies that a lack of reliable and valid scientific evidence in the field of rehabilitation has made policy-makers and practitioners question the effectiveness of these interventions [72,76,84]. Thus, with the participation of physiotherapy departments and scientific associations, it is necessary to take desirable measures to prepare quality and substantiated scientific evidence, such as clinical guidelines [85].

More patients are referred in the context of PHC, putting a high workload on the service providers [86,87]. In particular, due to the longer duration of physiotherapy services, physiotherapists’ time constraints and workload are more significant. This factor leads to long waiting lists for receiving these services, which could cause patient dissatisfaction and burnout among physiotherapists [88]. To improve this situation, studies have emphasized the necessity of a comprehensive and effective referral system [84]. By creating a suitable referral system, it is possible to manage the number of referrals, reduce the burden on service providers, and guarantee cohesion and continuity of the treatment process [89]. Non-adherence of the professions involved in PHC to clinical guidelines was another challenge mentioned in some studies [43,90,91]. Several clinical guidelines have been developed in recent years, but studies show that adherence is still low, and the gap between evidence and practice remains [90]. Various factors play a role in such conditions, such as lack of guideline trustworthiness, time constraints, the negative attitude of practitioners toward guidelines, and lack of continuous clinical training programs [[90], [91], [92]]. Therefore, it is necessary to develop effective strategies to address misconceptions and other common obstacles to the adoption of clinical guidelines by PHC team members to curb the evidence-practice gap. One approach can be increasing the ability of physiotherapists to benefit from scientific evidence, such as by searching for evidence and evaluating its quality.

In PHC, physiotherapists encounter patients who need complex care, which is one of their challenges [32,63]. These patients need physiotherapy services that may be beyond the abilities of physiotherapists working in PHC centers. Proper referral of these individuals to specialized centers can reduce the pressure on physiotherapists in PHC. Another interesting finding in this study was that physiotherapists stated that their employment in primary care carried the potential risk of isolating them from their professional peers [7,36,40]. In this regard, it seems that not having enough access to other team members and doing work alone causes a sense of isolation and fatigue for physiotherapists [93]. Such conditions are seen especially in low-income countries, where physiotherapists are very much on their own anyway, with little opportunity for professional exchange. To minimize such negative feelings, it is necessary to promote interprofessional collaborations along with increasing managerial support and mentorship. Language barriers were another challenge found in this study, as mentioned in other studies [94,95]. Differences in the languages of patients and therapists can cause problems in communication between them. Furthermore, our review of qualitative literature showed that physiotherapists need to develop their knowledge of psychology to meet the demands of primary care. Based on the available evidence, adopting bio-psychosocial approaches by practitioners could facilitate interpersonal interactions and improve the interventions’ effects, especially for people with musculoskeletal disorders, among whom the prevalence of psychological disorders is high [96,97]. In addition, physiotherapists working in PHC need to be familiar with assistive technology services so that they can provide these services to target groups as part of their duties [9,14]. It is also possible to use other rehabilitation professions, such as occupational therapists, who are more familiar with assistive technologies. Therefore, it is necessary to make changes in the educational curriculum of PT students to empower them as providers of PHC services. Being able to work flexibly, having communication skills, understanding different levels of health care systems, and having knowledge of psychology and pharmacology should be embedded in the educational curriculum of physiotherapist students [57].

Providing physiotherapy services in primary care settings requires the allocation of sufficient financial resources [51]. However, financing rehabilitation services, including physiotherapy, is often done through out-of-pocket payments, and government participation is insufficient [98]. This study found inadequate financial resources as one of the main obstacles to integrating physiotherapy services into primary care. Therefore, it is necessary to provide the financial resources needed to deliver these services in the context of primary care. Among the potential strategies, we can point out the provision of a package of physiotherapy services and their coverage by basic health insurance, mainly financed by government insurance funds [84]. The payment system in PHC has always been criticized [76,84,99]. Therefore, it is necessary to consider value-based and competitive payment mechanisms that provide sufficient incentives for providers to participate in this program [100]. This issue is critical in connection with physiotherapists because, in many countries, they work in private physiotherapy centers and often have a good income [9]. Therefore, it is imperative to consider a favorable service compensation mechanism to encourage them to attend PHC.

In relation to human resources, the findings showed that various challenges affect the performance of physiotherapists in PHC. Educational challenges were among the most common topics mentioned in several studies. According to the nature of PHC, physiotherapists must learn the necessary skills related to using clinical guidelines, managing patients with complex conditions, developing administrative skills, and using scientific evidence [101,102]. In other words, academic training and continuous training during work should align with the demands of the field of PHC [103]. An adequate opportunity should also be provided for physiotherapists on the path of personal and professional development to have sufficient motivation to attend this program [40]. On the other hand, the multiple roles in PHC have meant that physiotherapists can no longer perform their main tasks effectively, as their workload has increased significantly [23]. This issue highlights the need to clarify the tasks and scope of physiotherapists in PHC. In fact, by adopting such an approach, it is possible to reduce their work pressure and increase their satisfaction, making the expectations of physiotherapists a reality.

The lack of human resources in the field of rehabilitation, including physiotherapy, has always been a significant challenge [104,105]. Therefore, taking advantage of various strategies is necessary to provide enough physiotherapists to attend the PHC program. One of the strategies suggested in the studies is using mobile physiotherapy teams [106], which can be very effective for covering remote areas, especially when specialized physiotherapy is needed. Furthermore, some studies have suggested that local volunteers and trained assistants can provide a range of physiotherapy services to patients [22]. Countries such as China and Chile have adopted such approaches to provide physiotherapy services in the primary care context [22,107]. Telerehabilitation and other web-based services could also be used to facilitate service delivery to rural and remote areas [108], especially after the COVID-19 pandemic [109]. However, such approaches require internet infrastructure, which unfortunately does not exist in many rural areas, especially in developing and underdeveloped countries [110]. In addition, they need suitable infrastructure and facilities in PHC environments. The weakness of the infrastructure is one of the serious obstacles to providing physiotherapy services [111]. Strengthening the information systems and technological infrastructure in the field of PHC can facilitate the provision of various care services, including physiotherapy services.

### 4.1 Strengths and limitations

This systematic scoping review provides new information regarding the common barriers and facilitators of integrating physiotherapy into PHC, on which, to the best of our knowledge, no study has been done. To minimize potential biases in selecting and extracting the relevant data, at least two independent reviewers were involved. On the other hand, this study faces a number of limitations. First, by focusing on the qualitative studies, we may have missed some relevant findings reported in the quantitative studies. Second, we only included studies published in English, which could be a source of publication bias. Third, considering that many input studies have been conducted in high-resourced countries, the findings of this study should be used more carefully in the context of less-resourced countries. In future studies, it is necessary to examine the strategies for integrating physiotherapy services into PHC from the perspective of the different stakeholders. It is also necessary to provide practical solutions to facilitate the integration of this category of services using implementation science.

## 5 Conclusion

The integration of physiotherapy services into 10.13039/100012453PHC faces many barriers, including a lack of intra- and inter-professional collaboration, ineffective teamwork, time constraints, and workloads, lack of clarity over the role and knowledge of physiotherapists, poor knowledge of GPs about physiotherapy, undesirable referral mechanisms, inadequate funding resources, educational challenges, and inadequate equipment. However, there are several facilitators for integrating these services into 10.13039/100012453PHC, including employing effective advocacy strategies, empowering the leadership, improving intra- and inter-professional collaborations, strengthening teamwork, enhancing patients’ awareness about physiotherapy, improving the working culture, creating a desirable referral system, effectively communicating between patients and physiotherapists, clarifying the roles of involved professionals, increasing funding resources, providing comprehensive training, facilitating personal and professional development, providing management and mentorship support, organizing an electronic information system, and applying new technological innovations.

## 6 Ethics approval and consent to participate

The ethical committee of the Shiraz University of Medical Sciences approved the study previously (IR.SUMS.REC.1401.242). All methods were performed in accordance with relevant guidelines and regulations.

## 7 Consent for publication

Not applicable.

## Funding

None.

## Authors' contributions

Manal Etemadi, Maryam Hedayati, Masoud Behzadifar: Analyzed and interpret the data; Performed the experiments; Wrote the paper**.**

Barth Cornelia Anne, Parviz Mojgani, Kamran Bagheri Lankarani: Performed the experiments; Wrote the paper**.**

## Availability of data and materials

The datasets used and/or analyzed during the current study are available from the corresponding author on reasonable request.

## CRediT authorship contribution statement

**Shabnam ShahAli:** Conceptualization, Data curation, Formal analysis, Investigation, Methodology, Supervision, Validation, Visualization, Writing – original draft, Writing – review & editing. **Saeed Shahabi:** Conceptualization, Data curation, Formal analysis, Investigation, Methodology, Project administration, Resources, Supervision, Validation, Visualization, Writing – original draft, Writing – review & editing. **Manal Etemadi:** Conceptualization, Formal analysis, Validation, Writing - original draft, Writing - review & editing. **Maryam Hedayati:** Conceptualization, Formal analysis, Methodology, Writing – original draft, Writing – review & editing. **Barth Cornelia Anne:** Methodology, Writing – original draft, Writing – review & editing. **Parviz Mojgani:** Conceptualization, Formal analysis, Writing – original draft, Writing – review & editing. **Masoud Behzadifar:** Conceptualization, Data curation, Writing – original draft, Writing – review & editing. **Kamran Bagheri Lankarani:** Conceptualization, Formal analysis, Writing – original draft, Writing – review & editing.

## Declaration of competing interest

The authors declare that they have no known competing financial interests or personal relationships that could have appeared to influence the work reported in this paper.
