# Supplementary material for: Barriers and facilitators of integrating physiotherapy into primary health care settings: A systematic scoping review of qualitative research
Source: Heliyon. 2023 Oct 6;9(10):e20736. doi: 10.1016/j.heliyon.2023.e20736 (PMC10582494; doi:10.1016/j.heliyon.2023.e20736)
Supplement: Multimedia Component 2 [file mmc2.docx]

**Supplementary Table 1.** Search strings

| **Search strategy for PubMed** |
| --- |
| ((Physiotherapy[tiab] OR “Physical therapy”[tiab] OR “Physical Therapist”[tiab] OR “Physical Therapists”[tiab] OR (Therapist[tiab] AND Physical[tiab]) OR (Therapists[tiab] AND Physical[tiab]) OR Physiotherapists[tiab] OR Physiotherapist[tiab]) AND (“Primary health care”[tiab] OR (Care[tiab] AND “Primary Health”[tiab]) OR (“Health Care”[tiab] AND Primary[tiab]) OR “Primary Healthcare”[tiab] OR (Healthcare[tiab] AND Primary[tiab]) OR “Primary Care”[tiab] OR (Care[tiab] AND Primary[tiab])) AND (“Qualitative study”[tiab] OR “qualitative research” [tiab] OR qualitative[tiab] OR interview[tiab])) |
| **Search strategy for Scopus** |
| ((TITLE-ABS(Physiotherapy) OR TITLE-ABS(“Physical therapy”) OR TITLE-ABS(“Physical Therapist”) OR TITLE-ABS(“Physical Therapists”) OR (TITLE-ABS(Therapist) AND TITLE-ABS(Physical)) OR (TITLE-ABS(Therapists) AND TITLE-ABS(Physical)) OR TITLE-ABS(Physiotherapists) OR TITLE-ABS(Physiotherapist)) AND (TITLE-ABS(“Primary health care”) OR (TITLE-ABS(Care) AND TITLE-ABS(“Primary Health”)) OR (TITLE-ABS(“Health Care”) AND TITLE-ABS(Primary)) OR TITLE-ABS(“Primary Healthcare”) OR (TITLE-ABS(Healthcare) AND TITLE-ABS(Primary)) OR TITLE-ABS(“Primary Care”) OR (TITLE-ABS(Care) AND TITLE-ABS(Primary))) AND (TITLE-ABS(“Qualitative study”) OR TITLE-ABS(“qualitative research”) OR TITLE-ABS(qualitative) OR TITLE-ABS(interview))) |
| **Search strategy for Web of Science** |
| ((TS=(Physiotherapy) OR TS=(“Physical therapy”) OR (TS=(Therapist) AND TS=(Physical)) OR (TS=(Therapists) AND TS=(Physical)) OR TS=(Physiotherapists) OR TS=(Physiotherapist)) AND (TS=(“Primary health care”) OR (TS=(Care) AND TS=(“Primary Health”)) OR (TS=(“Health Care”) AND TS=(Primary)) OR TS=(“Primary Healthcare”) OR (TS=(Healthcare) AND TS=(Primary)) OR TS=(“Primary Care”) OR (TS=(Care) AND TS=(Primary))) AND (TS=(“Qualitative study”) OR TS=(“qualitative research”) OR TS=(qualitative) OR TS=(interview))) |

**Supplementary Table 2.** Excluded studies with exclusion reason

| Author (year) | Title | Reason for exclusion |
| --- | --- | --- |
| Al-Abbad et al. 2016 | The perception of physical therapy leaders in Saudi Arabia regarding physical therapy scope of practice in primary health care | No qualitative study |
| Bandong et al. (2018) | Referral to specialist physiotherapists in the management of whiplash associated disorders: Perspectives of healthcare practitioners | Not about PHC |
| Bastemeijer et al. (2021) | Patient values in physiotherapy practice, a qualitative study | Not about PHC |
| Bishop et al. (2015) | "lovely pie in the sky plans": A qualitative study of clinicians' perspectives on guidelines for managing low back pain in primary care in England | Not about PHC |
| Bodenheimer et al. (2021) | Integrating Physical Therapists Into Primary Care Within A Large Health Care System | No qualitative study |
| Bouma et al. (2021) | What affects the implementation of lifestyle interventions in patients with osteoarthritis? A multidisciplinary focus group study among healthcare professionals | Not about PHC |
| Brewer et al. (2021) | Physiotherapists’ experiences with and perspectives on implementing an evidence-based, chronic pain self-management programme in primary health care: A qualitative study | Not about PHC |
| Budtz et al. (2022) | Primary Care Physical Therapists' Experiences When Screening for Serious Pathologies Among Their Patients: A Qualitative Study | Not about PHC |
| Byles et al. (2002) | The experiences of non-medical health professionals undertaking community-based health assessments for people aged 75 years and over | Not about PHC |
| Carpenter (1994) | The experience of spinal cord injury: the individual's perspective--implications for rehabilitation practice | Not about PHC |
| Cederbom et al. (2022) | A qualitative study exploring physical therapists’ views on the Otago Exercise Programme for fall prevention: a stepping stone to “age in place” and to give faith in the future | Not about PHC |
| Chan et al. (2010) | Finding common ground? Evaluating an intervention to improve teamwork among primary health-care professionals | Not just physiotherapy services |
| Charumbira et al. (2021) | Physiotherapists’ awareness of risk of bone demineralisation and falls in people living with HIV: a qualitative study | Not about PHC |
| Charumbira et al. (2021) | Health system challenges affecting falls prevention in persons living with HIV: Perspectives from physiotherapists in four sub-Saharan regions | Not about PHC |
| Che Hasan et al. (2020) | Perspectives of ESCAPE-Pain Programme for Older People With Knee Osteoarthritis in the Community Setting | Not about PHC |
| Christiansen et al. (2020) | “It ... Doesn’t always make it [to] the top of the list” Primary care physicians’ experiences with prescribing exercise for knee osteoarthritis | Not just physiotherapy services |
| Clemence & Seamark (2003) | GP referral for physiotherapy to musculoskeletal conditions - A qualitative study | Not about PHC |
| Corbett et al. (2009) | GP attitudes and self-reported behaviour in primary care consultations for low back pain | Not just physiotherapy services |
| Cunningham et al. (2022) | First contact physiotherapists’ perceptions and experiences of practice-based small group learning in NHS Scotland: a qualitative study | Not just about PHC |
| Danielsson et al. (2013) | To sense and make sense of anxiety: Physiotherapists' perceptions of their treatment for patients with generalized anxiety | Not just about PHC |
| Danielsson et al. (2016) | “Crawling out of the cocoon”: Patients’ experiences of a physical therapy exercise intervention in the treatment of major depression | Not just about PHC |
| Davidson et al. (2022) | Perspectives of emergency department clinicians on the challenges of addressing low back pain in the emergency setting: A qualitative study | Not just about PHC |
| de Rooij et al. (2020) | Evaluation of an educational course for primary care physiotherapists on comorbidity-adapted exercise therapy in knee osteoarthritis: an observational study | Not qualitative study |
| De Souza et al. (2012) | Integral health assistance: A look to physiotherapy from the point of view of the Family Health Team | Not just about PHC |
| Defriez et al. (2003) | The perception of the current provision of care for multiple sclerosis sufferers in the community | Not just about PHC |
| Demont et al. (2020) | Impact of models of care integrating direct access to physiotherapy in primary care and emergency care contexts in patients with musculoskeletal disorders: A narrative review | Not just about PHC |
| Frost et al. (2022) | Advanced Practice Physiotherapists and the implementation of the JIGSAW-E model for the management of osteoarthritis in Scottish primary care settings: a qualitative case study | Not just about PHC |
| Egerton et al. (2017) | A systematic review and evidence synthesis of qualitative studies to identify primary care clinicians' barriers and enablers to the management of osteoarthritis | Not qualitative study |
| Eisen et al. (2021) | The Needs and Experiences of Patients on Pain Education and the Clinical Reasoning of Physical Therapists Regarding Cancer-Related Pain. A Qualitative Study | Not just about PHC |
| Ellis & Connell (2001) | Factors determining the current use of physiotherapy assistants: Views on their future role in the South and West UK region | Not just about PHC |
| Enthoven et al. (2021) | Patients’ experiences of the BetterBack model of care for low back pain in primary care–a qualitative interview study | Not just physiotherapy services |
| Farquhar et al. (2020) | Mechanisms to achieve a successful rural physiotherapy public-private partnership: a qualitative study | Not just about PHC |
| Feldthusen et al. (2022) | Experience of co-creation of a health plan and support for sustainable physical activity among people with chronic widespread pain: a qualitative interview study in Sweden | Not just about PHC |
| Ferreira et al. (2018) | Staff and patients have mostly positive perceptions of physiotherapists working in emergency departments: a systematic review | Not qualitative study |
| Fisher (2020) | Physical therapy in the emergency department: Surveying primary care practitioners' perceptions and recommendations | Not just about PHC |
| Foley et al. (2020) | A qualitative study of the dementia-care experiences and educational needs of physiotherapists in the Republic of Ireland | Not just about PHC |
| Foster et al. (2020) | Stratified versus usual care for the management of primary care patients with sciatica: the SCOPiC RCT | Not just about PHC |
| Frygner-Holm et al. (2021) | Physical therapists' experiences of learning and delivering a complex behavioral medicine intervention to adolescents with pain | Not just about PHC |
| Gard (2007) | Factors important for good interaction in physiotherapy treatment of persons who have undergone torture: A qualitative study | Not just about PHC |
| Geerars et al. (2021) | Decision-Making on Referral to Primary Care Physiotherapy After Inpatient Stroke Rehabilitation | Not just physiotherapy services |
| Gill et al. (2019) | Consumer preferences regarding physiotherapy practitioners and nurse practitioners in emergency departments - a qualitative investigation | Not just about PHC |
| Greenstein et al. (2016) | Improving physiotherapy services to Indigenous children with physical disability: Are client perspectives missed in the continuous quality improvement approach? | Not just about PHC |
| Griffiths et al. (2004) | Interdisciplinary teamwork in the community rehabilitation of older adults: An example of flexible working in primary care | Not just physiotherapy services |
| Gustavsson & Eriksson-Crommert (2020) | Physiotherapists' and midwives' views of increased inter recti abdominis distance and its management in women after childbirth | Not just about PHC |
| Gyllensten et al. (1999) | Interaction between patient and physiotherapist: a qualitative study reflecting the physiotherapist's perspective | Not just about PHC |
| Håkstad et al. (2018) | A qualitative study of clinical reasoning in physiotherapy with preterm infants and their parents: Action and interaction | Not just about PHC |
| Hannane et al. (2019) | Asthma patients' perception on their care pathway: a qualitative study | Not just physiotherapy services |
| Hendriks et al. (1996) | Experiences with physiotherapists' consultation: Results of a feasibility study | Not just about PHC |
| Hills & Kitchen (2007) | Satisfaction with outpatient physiotherapy: Focus groups to explore the views of patients with acute and chronic musculoskeletal conditions | Not just about PHC |
| Holopainen et al. (2022) | An adventurous learning journey. Physiotherapists' conceptions of learning and integrating cognitive functional therapy into clinical practice | Not just about PHC |
| Hordvik et al. (2021) | Physiotherapists´ experiences with older adults´ rehabilitation trajectory after hip fracture: A qualitative study in Western Norway | Not just about PHC |
| Hughes et al. (2022) | Clinician views on optimism and empathy in primary care consultations | Not just physiotherapy services |
| Igwesi-Chidobe et al. (2020) | Evidence, theory and context: Using intervention mapping in the development of a community-based self-management program for chronic low back pain in a rural African primary care setting-the good back program | Not just physiotherapy services |
| Irgens et al. (2020) | Variations in physiotherapy practice in neurological rehabilitation trajectories -an explorative interview and observational study | Not qualitative study |
| Josephson & Bülow (2014) | Utilization of patient resources in physiotherapy interventions: Analysis of the interaction concerning non-specific low back pain | Not just about PHC |
| Karstens et al. (2015) | General practitioners views of implementing a stratified treatment approach for low back pain in Germany: A qualitative study | Not just about PHC |
| Kasper et al. (2022) | Primary Care as a practice and learning setting in the education of physiotherapists in Brazil: the perception of students, professionals and users | Not just about PHC |
| Keen et al. (2018) | Physiotherapy practice in pulmonary hypertension: physiotherapist and patient perspectives | Not just about PHC |
| Knoop et al. (2020) | Is a model of stratified exercise therapy by physical therapists in primary care feasible in patients with knee osteoarthritis?: a mixed methods study | Not qualitative study |
| Kyle et al. (2020) | What helps and hinders the provision of healthcare that minimises treatment burden and maximises patient capacity? A qualitative study of stroke health professional perspectives | Not just physiotherapy services |
| Liem et al. (2022) | Consensus-based recommendations on communication and education regarding primary care physical therapy for patients with systemic sclerosis | Not just about PHC |
| Mackenzie et al. (2020) | Fall prevention in primary care using chronic disease management plans: A process evaluation of provider and consumer perspectives | Not just physiotherapy services |
| Mackenzie & Clifford (2018) | Perceptions of primary health staff about falls prevention in primary care settings in the west of Ireland | Not just physiotherapy services |
| Mårtensson (2001) | Rehabilitation of Patients with Chronic Pain in Primary Health Care | Not just physiotherapy services |
| Pinnington et al. (2004) | An evaluation of prompt access to physiotherapy in the management of low back pain in primary care | Not just about PHC |
| Oosman et al. (2017) | Enhancing Access to Physical Therapy Services for People Experiencing Poverty and Homelessness: The Lighthouse Pilot Project | Not just about PHC |
| Middlebrook & Mackenzie (2011) | The Enhanced Primary Care program and falls prevention: Perceptions of private occupational therapists and physiotherapists | Not just physiotherapy services |
| Locke et al. (2019) | Musculoskeletal (MSK) practitioners in primary care: an evaluation of a MSK core capabilities framework and review process | Not just about PHC |
| Kelly et al. (2017) | Health practitioners’ perceptions of adopting clinical prediction rules in the management of musculoskeletal pain: a qualitative study in Australia | Not just about PHC |
| Håkstad et al. (2010) | Parents’ Perceptions of Physiotherapy in Primary Health Care With Preterm Infants: Normalization, Clarity and Trust | Not just about challenges of PHC |
| Gunn & Goding (2009) | Continuing Professional Development of physiotherapists based in community primary care trusts: a qualitative study investigating perceptions, experiences and outcomes | Not just about PHC |
| Coyle & Gill (2017) | Acceptance of primary practitioner physiotherapists in an emergency department: A qualitative study of interprofessional collaboration  within workforce reform | Not just about PHC |
| Brice-Leddy et al. (2020) | Enabling AccesstoRehabilitationinAcuteCare: Exploring Physiotherapists’ and Occupational Therapists’ Perspectives onPatientCareWhen Assistants BecomethePrimaryTherapyProviders | Not just about PHC |
| Bernhardsson et al. (2019) | A preference for dialogue: exploring the influence of patient preferences on clinical decision making and treatment in primary care physiotherapy | Not just about challenges of PHC |
| Bernhardsson et al. (2017) | “In the physio we trust”: A qualitative study on patients’ preferences for physiotherapy | Not just about PHC |
| Bastiaens et al. (2021) | Identifying goals, roles and tasks of extended scope physiotherapy in Dutch primary care- an exploratory, qualitative multi-step study | Not just about challenges of PHC |
| Alhowimel et al. (2021) | Management of Low back pain in Saudi Arabia healthcare system. A Qualitative Study | Not just about challenges of PHC |
| Saunders et al. (2020) | Patients’ and clinicians’ perspectives on a ‘fast-track’ pathway for patients with sciatica in primary care: qualitative findings from the SCOPiC stratified care trial | Not just physiotherapy services |
| Saunders et al. (2021) | First Contact Practitioners’ (FCPs) and General Practitioners’ Perceptions Towards FCPs Delivering Vocational Advice to Patients with Musculoskeletal Conditions: A Qualitative Investigation of the Implementation Potential of the I‑SWAP Initiative | Not just physiotherapy services |
| Roberts et al. (2003) | A national qualitative survey of community-based musculoskeletal services in the UK | Not just physiotherapy services |
| Roberts et al. (2002) | Improving the quality of care of musculoskeletal conditions in primary care | Not just physiotherapy services |
| Rasmussen-Barr et al. (2021) | Are patient reported outcome measures (PROMs) useful in low back pain? Experiences of physiotherapists in primary health care in Sweden | Not just about challenges of PHC |
| Ramírez-Vélez et al. (2015) | Barriers against incorporating evidence-based practice in physical therapy in Colombia: current state and factors associated | Not just about challenges of PHC |
| Neher et al. (2021) | Preparedness to Implement Physical Activity and Rehabilitation Guidelines in Routine Primary Care Cancer Rehabilitation: Focus Group Interviews Exploring Rehabilitation Professionals’ Perceptions | Not just about challenges of PHC |
| Mengshoel et al. (2021) | ‘It takes time, but recovering makes it worthwhile’- A qualitative study of long-term users’ experiences of physiotherapy in primary health care | Not just about challenges of PHC |
| Meerhoff et al. (2021) | Exploring the perspective of patients with musculoskeletal health problems in primary care on the use of patient-reported outcome measures to stimulate quality improvement in physiotherapist practice; a qualitative study | Not just about challenges of PHC |
| Mackenzie et al. (2019) | Fall prevention in primary care using chronic disease management plans: A process evaluation of provider and consumer perspectives | Not just physiotherapy services |
| Mullan et al. (2023) | The experiences of physiotherapy independent prescribing in primary care: implications for practice | Not about integrating physiotherapy services into PHC |
| Lundin & Bergenheim (2020) | Encountering suicide in primary healthcare rehabilitation: the experiences of physiotherapists | Not just about challenges of PHC |
| Holdsworth et al. (2008) | Physiotherapists’ and general practitioners’ views of self-referral and physiotherapy scope of practice: results from a national trial | Not just about challenges of PHC |
| Håkstad et al. (2018) | A Qualitative Study of Clinical Reasoning in Physiotherapy with Preterm Infants and Their Parents: Action and Interaction. | Not just about challenges of PHC |
| Geraghty et al. (2020) | Exploring Patients’ Experiences of Internet-Based Self-Management Support for Low Back Pain in Primary Care | Not just physiotherapy services |
| Gard et al. (2000) | Physical Therapists’ Emotional Expressions in Interviews about Factors Important for Interaction with Patients | Not just about challenges of PHC |
| Gard (2007) | Factors important for good interaction in physiotherapy treatment of persons who have undergone torture: A qualitative study | Not just about challenges of PHC |
